# Multidimensional Deprivations and Associated Factors Among Older Adults in Urban Geographies of Nigeria: Implications for Poor Health Outcomes in Later Life

**DOI:** 10.3389/ijph.2024.1606572

**Published:** 2024-03-13

**Authors:** Jacob Wale Mobolaji

**Affiliations:** Department of Demography and Social Statistics, Obafemi Awolowo University, Ile-Ife, Nigeria

**Keywords:** older adults, multidimensional deprivation, poverty, standard of living, socioeconomic status, multilevel analysis

## Abstract

**Objectives:** Many urban-dwelling older Nigerians are multidimensionally deprived and are unable to meet their daily financial, nutritional, and healthcare needs. This has implications for their health outcomes, yet it has been under-researched. This study assessed the multidimensional deprivation index (MDI) of urban-dwelling older Nigerians and the associated factors.

**Methods:** The study analysed a weighted sample of 5,225 older persons aged ≥60 years from Nigeria’s Demographic and Health Survey, 2018. MDI was estimated, and associations were examined using a multilevel multinomial logistic regression model.

**Results:** Nationally, 75% of the older persons were multidimensionally deprived, with 27% severely deprived. Women (36%) were more severely deprived than men (20%). Those in the Northern regions (38%–40%) were the most deprived. Higher MD risk was associated with female gender and older ages ≥70–79 years. Conversely, lower risk was associated with households headed by family and residence in educated communities. Community variation accounts for 10.4% and 35.9% of the MD and severe MD risks, respectively.

**Conclusion:** This study suggests socioeconomic interventions that address gender disparities and target highly deprived regions, with consideration for individual and community characteristics.

## Introduction

Old age is characterized by emerging needs and difficulties due to decreasing physical functioning and health deterioration. In Nigeria, the population of older adults is rapidly rising with the associated health challenges [1, 2]. In the next 10 years, the proportion of the population moving into the older age group will be very alarming. The Nigerian government recently approved the first national policy on older people [3], however, the evidence and content provided left more to be desired. Evidence suggests that about 70% of the older Nigerians are vulnerable and poor [4], with the inability to meet their daily financial, nutritional, and healthcare needs; yet little is known about the complexities and dimensions of their poverty and vulnerability. Multiple and co-existing deprivations in the socioeconomic and healthcare resources accentuate the risk of poor health outcomes and lower life expectancy in later life.

The concept of multidimensional deprivation aims at capturing poverty, otherwise referred to as deprivation, in all its forms as conceived in the sustainable development goal (SDG) 1 [5]. The concept relates to the measures of household deprivations in multiple dimensions and their corresponding indicators which include health (measured by child death and nutritional status), education (years of formal education, and child’s school enrolments) and standard of living (sanitation, water, floor, household assets, cooking fuel, electricity) [6–8]. While the multidimensional approach has been adapted to different population sub-groups across the world, especially children and older people [9–12], little has been done in Nigeria. The few multidimensional poverty studies in Nigeria have either focused on the general household or children, with little/no research attention to older adults.

Existing evidence shows that residents of urban settings are socioeconomically better than those in rural geographies [13, 14]. However, there are unassessed disparities and nuances in urban settings. Urban geographies in many developing countries, especially in Nigeria, are susceptible to alarming crime rates, poverty, high cost of living, environmental hazards and socioeconomic inequalities [15–17]. Poverty in the urban setting is linked to the worsening environmental degradation and poor socioeconomic infrastructure coupled with poor governance and institutional deficiencies [18–21]. This study argues that the socioeconomic condition of older urban residents, especially vulnerable older adults residing in slums, is worse than rural dwellers. Although growing old and maintaining wellbeing and resilience in such settings could be challenging, research evidence teasing out the dimensions and intensity of poverty realities of the older residents is lacking in Nigeria. This study examines the extent of multidimensional deprivations and associated factors among older people in Nigeria. The findings from this study will serve as a policy guide for improving the welfare and wellbeing of older people in the country.

## Methods

### Data Sources and Sample Design

The study employed secondary analysis of household members’ data from Nigerian Demographic and Health Surveys (DHS), 2018. The household version of NDHS is nationally representative and contains demographic, socioeconomic and health information about individuals in the selected households. A stratified two-stage cluster design was used to select households from 904 clusters. A detailed description of the sample design of the survey was already published elsewhere [22]. Information on individual socio-demographic characteristics and household living conditions of a weighted sample of 5,225 older members of the household aged 60 years and above were extracted and used for this study. The sample was weighted to correct the possible sampling errors during the data collection.

### Variables and Measures

The multidimensional deprivation index (MDI), the outcome variable for this study, was measured using five dimensions: health, education, standard of living, information and communication, and empowerment. The five dimensions were computed from 15 indicators including the experience of functional limitations; level of education; official language literacy; sources of drinking water; type of toilet facilities regarded as sanitation; type of cooking fuel; access to electricity; type of housing materials; access to sources of information such as television and radio sets; access to means of communication such as mobile phone or landlines; ownership of agricultural land, livestock or farm animals; access to means of mobility; and access to a bank account. Existing studies have used different dimensions and indicators for different population sub-groups. For instance, most authors have used dimensions like disability, standard of living, education and housing burden [9–12]. The Nigerian National Multidimensional Poverty Index 2021 used education, unemployment, health in terms of nutrition food security and time to healthcare, and living standard and poverty. However, the data is not publicly available at the time of this study. The dimensions and indicators used in this study were adapted from the existing studies identified above and based on data availability.

Each of the indicators was assigned a weight within its corresponding dimension, while all dimensions *d* were given equal relative weight *w*. The multidimensional deprivation (MD) score *m* for *ith* respondent is the weighted sum of all deprivations such that

$$m_{i}=\sum_{j=1}^{d}w_{ij}=1$$ (1)

Deprivation score which lies between zero and one (0–1) was assigned to each respondent. As the number of deprivations increases, the score of the respondent increases to a maximum of 1 which indicates extreme deprivation, while zero represents no deprivation. Please see the details of the variables, measures and corresponding weights in Table 1. Although scholars have used different weighting strategies including multiple cluster analysis [23] and composite scoring of 1 for the deprived and 0 for non-deprived persons in each indicator [12], this study used the latter approach which is the most popularly used weighting method in measuring multidimensional deprivations.

**TABLE 1:** Dimensions, indicators and measurements of multidimensional deprivation index (Nigeria, 2023).

Dimensions	Indicators	Deprivation descriptions	Weights	Total weights
Health	Functional limitations	^a^Has difficulty in any of seeing, hearing, walking, talking, memory, dressing self	1/5	1/5
Education	Level of education	Has not completed at least primary school	1/10	1/5
	Official language literacy	Does not understand English (official) language	1/10	
Living standard	Drinking water	Unimproved water	1/25	1/5
	Sanitation	Unimproved sanitation	1/25	
	Cooking fuel	unimproved cooking fuel	1/25	
	Electricity	No electricity	1/25	
	housing	Unimproved floor/roof/wall	1/25	
Information, Communication and Companionship	Information	No access to radio or TV	1/15	1/5
	Communication	No access to telephone	1/15	
	Companionship	Living alone	1/15	
Empowerment	Ownership of agricultural land, livestock, farm animals, cattle/cow/bulls/donkeys	^b^Has less than 1 ha of land or zero horse/camel/donkey or less than 2 cattle/cow/bulls or less than 8 goats/sheep/pigs or less than 30 poultry birds	1/15	1/5
	Mobility	^b^Has no car/truck or bike/scooter or animal-drawn cart or boat with a motor, motorized tricycle	1/15	
	Bank account	No bank account	1/15	

^a^ Difficulty in any of the listed items means deprivation.

^b^ Ownership of any of the listed items is not deprived.

A deprivation threshold (*t*) also referred to as the cut-off value which is the percentage of the indicators in which a respondent is deprived was set, such that persons below the *t* are considered not deprived at the *t* dimension. Persons who are deprived in 20% of the 15 indicators are deprived at *t* = 1; those that were deprived in 40% of the indicators are deprived at *t* = 2; and deprivations in 60%, 80% and 100% of the indicators are deprived at *t* = 3, *t* = 4 and *t* = 5, respectively. A cut-off value of 40% (*t* = 2) was regarded as MD in this study. Though some studies have used a cut-off point of *t* = 1 for MD, the choice of cut-off *t* = 2 was based on the high poverty level in the country. Available evidence suggests that raising the cut-off point helps to adjust for the poverty level in poverty-tolerant contexts of low- and middle-income countries (LMIC) [11].

Overall, multidimensionally deprived persons were identified from the MDI. Persons who were deprived in less than 20% of the indicators were categorised as not deprived; individuals who were deprived in 20%–39.9% of the indicators were categorised as being vulnerable to MD; while persons deprived in 40%–59.9% of the indicators were referred to as being multidimensionally deprived; and those that were deprived in ≥60% of the indicators were categorised as having severe MD. For inferential statistical tests and multivariable analysis, the MD levels were recoded into three: no MD (merging not deprived with vulnerable to MD) with code = 0, multidimensionally deprived (code = 1) and severe MD (code = 2).

The independent variables are the individual socio-demographic, household and community characteristics of the respondents. The sociodemographic characteristics include age, sex, marital status, sex and age of their household head. The community variables include community level of education and poverty level (each categorised as low, medium and high), and region of residence. The community variables were generated from the existing variables, except the region of residence.

### Data Analysis

The data for this study was analysed in two sections using Stata version 15.1 software. The first section addressed the estimates of the multidimensional deprivation index while the second section focused on the predictors of multidimensional deprivation among older adults in Nigeria using the multilevel multinomial logistic regression model.

#### Multidimensional Deprivation Estimates

The multidimensional deprivation measures including the headcount ratio (*H*), intensity (*I*), relative shares of MD, and the adjusted headcount ratio (R_0_) as well as their gender and regional disparities were estimated and presented in Tables 2–4. According to Alkire and Foster [8], the headcount ratio *H* indicating the proportion of respondents with multiple deprivations is given by

$$H=\frac{p}{n}$$ (2)

Where *p* is the number of respondents who are multidimensionally deprived. This is the incidence of MD in the population while *n* is the total population. In this study, the headcount ratio of multidimensionally deprived older persons was estimated using the above formula, expressed in percentages and presented at various cut-off values. The *H* of older Nigerians were estimated for those who were vulnerable to multidimensional deprivation (t = 1); multidimensionally deprived (t = 2); and those with severe multidimensional deprivation (t ≥ 3). The estimates were also obtained by gender and region of residence.

**TABLE 2:** Unweighted and weighted distribution of the respondents (Nigeria, 2023).

Sociodemographic characteristics	Unweighted sample		Weighted sample	
	n	%	n	%
Age group				
<70	2,805	57.6	3,037	58.1
70–79	1,429	29.4	1,535	29.4
80+	633	13.0	653	12.5
Sex				
Male	2,638	54.2	2,877	55.1
Female	2,229	45.8	2,348	44.9
Marital Status				
Single	26	0.5	26	0.5
Married	3,131	64.3	3,416	65.4
Divorced	79	1.6	77	1.5
Widowed	1,631	33.5	1706	32.6
Household head				
Self (respondent)	3,716	76.3	3,961	75.8
Someone else	1,151	23.7	1,264	24.2
Region of residence				
North Central	610	12.5	492	9.4
North East	359	7.4	382	7.3
North West	641	13.2	784	15.0
South East	1,307	26.8	1,241	23.7
South South	569	11.7	583	11.2
South West	1,381	28.4	1743	33.4
Total	4,867	100.0	5,225	100.0

**TABLE 3:** Multidimensional deprivation index among older adults (Nigeria, 2023).

	Overall			Male			Female		
Cut off points	R_0_	H	I	R_0_	H	I	R_0_	H	I
Vulnerable to MD (t = 1)	0.072	22.7	31.9	0.083	26.4	31.6	0.058	17.8	32.5
MD (t = 2)	0.244	48.8	49.9	0.250	50.6	49.5	0.228	45.3	50.4
Severe MD (t = 3)	0.184	26.6	69.4	0.137	20.0	68.5	0.253	36.1	70.1

Note: MD, multidimensional deprivation; vulnerable to MD: respondents deprived in 20%–39.9% of the indicators; MD: deprived in 40%–59% of the indicators; severe MD: deprived in ≥60% of the indicators; H multidimensional headcount ratio at each cut-off point; I intensity of deprivation among the poor; R_0_ Adjusted headcount ratio (multiplication of H and I).

**TABLE 4:** Subnational decomposition of multidimensional deprivations among older adults (Nigeria, 2023).

	Overall			Male			Female		
Cut off points	R_0_	P%	I%	R_0_	P%	I%	R_0_	P%	I%
North Central									
Vulnerable to MD	0.069	21.8	31.6	0.132	27.3	48.2	0.080	14.6	54.7
MD	0.250	50.3	49.8	0.277	50.6	54.8	0.294	50	58.7
Severe MD	0.181	25.9	69.9	0.131	18.9	69.1	0.247	35	70.5
North East									
Vulnerable to MD	0.046	13.9	32.8	0.001	16.3	0.538	0.057	9.6	59
MD	0.231	45.7	50.5	0.287	49.5	57.9	0.237	38.4	61.8
Severe MD	0.278	40.1	69.4	0.233	33.8	69	0.363	52	69.8
North West									
Vulnerable to MD	0.062	19.6	31.5	0.116	22.5	51.5	0.079	13.5	58.2
MD	0.208	41.7	49.8	0.253	44.0	57.6	0.228	36.7	62
Severe MD	0.263	37.9	69.4	0.222	32.3	68.6	0.351	49.8	70.4
South East									
Vulnerable to MD	0.068	21	32.5	0.128	26.1	48.9	0.090	16.4	55
MD	0.246	48.8	50.4	0.281	51.0	55.1	0.278	46.8	59.4
Severe MD	0.197	28.2	69.9	0.133	19.4	68.6	0.255	36.2	70.5
South South									
Vulnerable to MD	0.092	29.5	31.1	0.139	31.9	43.6	0.134	26.9	49.9
MD	0.238	48.9	48.7	0.266	53.0	50.2	0.255	44.6	57.1
Severe MD	0.116	17.0	68.3	0.055	8.2	67.4	0.180	26.3	68.6
South West									
Vulnerable to MD	0.079	24.8	32	0.139	29.8	46.6	0.104	19.6	53.2
MD	0.249	50	49.7	0.286	53.6	53.4	0.268	46.1	58.2
Severe MD	0.161	23.3	69.2	0.094	13.9	67.6	0.233	33.3	69.9

Note: MD, multidimensional deprivation; vulnerable to MD: respondents deprived in 20%–39.9% of the indicators; MD: deprived in 40%–59% of the indicators; severe MD: deprived in ≥60% of the indicators; P proportion considered multidimensionally deprived at each cut-off point; I intensity of deprivation among the poor; R_0_ Adjusted headcount ratio (multiplication of P and I).

In addition, the Intensity *I* of multidimensional deprivation which is the average share of deprivation among the poor is another measure of MD, based on Alkire and Foster’s formula

$$I=\frac{\sum_{i=1}^{n}m_{i}\left(t\right)}{d\times p}$$ (3)

Where *m* is the weighted sum of the MD score as indicated in Eq. 1; *t* is the chosen MD threshold, *d* is the total number of dimensions being considered and *p* represents the incidence of deprivation at the target threshold as shown in Eq. 2. The formula in Eq. 3 above was used to estimate the intensity of MD among older persons in Nigeria at national and subnational levels and by gender.

The adjusted headcount ratio *R*_*o*_ was also estimated for various levels of multidimensional deprivation among older Nigerians. The *R*_*o*_ was obtained by the product of the headcount ratio and intensity of MD, as shown in Eq. 4 below.

$$R_{0}=H\;X\;I$$ (4)

Furthermore, the relative share of each multidimensional deprivation of each of the dimensions was also estimated at both national and subnational levels, and by gender.

#### Multilevel Multinomial Logistic Regression Model

The associated factors of multidimensional deprivation were examined using multilevel multinomial logistic regression (MMLR), encompassing both fixed-effect and random effects models. The outcome of an MMLR consists of *k* categories indexed using positive integers *1, 2, 3,* … , *k*. Given the Generalized Bernoulli distribution of the outcome variable (multidimensional deprivation: no MD [k = 1], multidimensionally deprived [k = 2] and severe MD [k ≥ 3]) in this study, the probability of observing each category *k* is denoted as πk. Taking *“*k = 1*”* as the reference category to establish a baseline against which other categories are compared, the model comprises K − 1 equations that analyse and contrast the odds of exhibiting the category *k* instead of the reference category.

The MMLR model is a mixed Generalized Linear Model (GLM) with linear predictors [24], generally given by Eq. 5 below

$$\gamma_{ij}=\alpha^{k}{{+\beta }^{k}}^{\prime }x_{ij}+{\xi_{j}}^{k}+{\delta_{ij}}^{k}$$ (5)

and multinomial logit link of

$$q\;\left(Y_{ij}=k\;\left|x_{ij},\xi_{ij},\delta_{ij}\right.\right)=\frac{{e^{\gamma_{ij}}}^{k}}{1+\sum_{l=2}^{K}{e^{\gamma_{ij}}}^{l}}$$ (6)

Where *k = 1, 2, … , K* represent the categories of the outcome variable (multidimensional deprivation); *j = 1, 2, … , J* represent the cluster (community) and *i = 1, 2, … , n*_*j*_ represent the respondents of the *jth* cluster.

Incorporating multilevel modelling into the multinomial logistic regression in Eqs 5, 6 allows variability in the probabilities of experiencing the *k* category across individuals. In each of the sub-equations, a random effect, represented by a varying intercept, is introduced. As described by Koster and McElreath [25], this varying intercept enables individuals to have either increased or decreased odds of being observed in category k relative to the reference category. The log-odds of ith individual experiencing other categories of K relative to the reference category is given as

$$\log\left(\frac{\gamma_{1ij}}{\gamma_{0ij}}\right)=\alpha_{1ij}+u_{1i}$$ (7)

$$\log\left(\frac{\gamma_{2ij}}{\gamma_{0ij}}\right)=\alpha_{2ij}+u_{2i}$$ (8)

$$\left[\begin{array}{c} u_{1i}\\ u_{2i} \end{array}\right]∼\text{Normal }\left(0,\pi_{u}\right):\pi_{u=}\left[\begin{array}{c} \sigma_{u1}^{2}\\ \sigma_{u1,2}\;\sigma_{u1,2}^{2} \end{array}\right]$$

$$\gamma_{0}+\gamma_{1}+\gamma_{2}=1$$ (9)

Where $\alpha_{1ij}$ and $\alpha_{2ij}$ are the intercepts that distinguish the second category in Eq. 7 and third category in Eq. 8 from the reference category, while *u*_1i_ and *u*_2i_ denote the individual-level random effects. These random effects are assumed to follow a multivariate normal distribution with zero means and a consistent 2 × 2 variance-covariance matrix. For brevity, only the intercept and random effects equations are presented. The sum of the proportion of individuals experiencing each k category equals 1 as shown in Eq. 9.

The above analytical approach was implemented by incorporating the logit link function of the GLM via the *gsem* Stata command. The analysis comprised four distinct nested models, including the empty model (comprising solely the outcome variable), model 1 (constructed solely with individual-level variables), model 2 (constructed solely with community-level variables), and model 3 (incorporating both individual and community-level variables). The optimal model selection for final result interpretation was determined through log-likelihood and Akaike’s information criteria.

The adjusted Relative Risk Ratio (RRR) with a 95% Confidence Interval (CI) was reported, and variables demonstrating a *p*-value of less than 0.05 in the multivariable analysis were identified as significant predictors of multidimensional deprivation (both for MD and severe MD). In random-effects analysis, the between-community variations in multidimensional deprivation were assessed using the Variance Partition Coefficient (VPC) otherwise referred to as Intraclass Correlation Coefficient (ICC) and Proportional Change in Variance (PCV). These measures provided insights into the extent of variation attributed to communities in the context of multidimensional deprivations.

Meanwhile, a multicollinearity test was conducted among explanatory variables using the Variance Inflation Factor (VIF). The results indicated the absence of multicollinearity, with VIF values ranging from 1.01 to 4.85 and a mean VIF of 2.35, except for a category of community-level poverty (VIF = 5.34). The recommended threshold for multicollinearity is a value greater than 5 to 10 [26, 27].

## Results

The results in Table 2 show the unweighted and weighted distribution of older persons selected for this study. Based on the weighted sample, more than half of them were males (55.1%) and 60–69 years old (58.1%). Although nearly two-thirds were married, a substantial proportion of them were widowed (32.6%). At least three-quarters of the respondents were household heads. A higher proportion of the total weighted sample was selected from the South West (33.4%) and South East (23.7%) while North East (7.3%) and North Central (9.4%) were the least represented.

Based on the estimate of the multidimensional deprivation index of older persons in Nigeria, over 75% of older Nigerians living in urban areas are multidimensionally deprived, with 27% classified as being in severe multidimensional deprivation (Table 3). An additional 23% of the people, about 2 million older persons, were vulnerable to multidimensional deprivation. The intensity of deprivation, which is the average poverty score among the multidimensionally deprived, indicates that the severely deprived people are deprived in over 69% of the indicators while the multidimensionally deprived had an average of 50% deprivation score. The MDI, which is the proportion of the population that is multidimensionally deprived adjusted by the intensity of the deprivation, is 0.244 while for those severely deprived is 0.184.

More specifically, the majority of the older persons lived in unimproved houses (83%), with no access to electricity (77%), drinking unimproved water (71%), illiterate of the national official language of communication (76%), had no means of mobility (66%) with health challenge (60%). Just about half (52%) had below secondary education. Less than half of the respondents were deprived of other indicators (result not shown).

There are wide gender inequalities in multidimensional poverty levels in Nigeria. For instance, while only 20% of the older men were severely deprived with the intensity of 68%, over 36% of their women counterparts had the same severity of deprivation. Thus, the MDI for women (0.253) was almost double that of men (0.137).

The subnational decomposition of the analysis indicates that the share of older persons multidimensionally deprived in at least 40% of the indicators (t = 2) in some regions was higher than the national rate, especially the North East (86%) and the North West (80%) (Table 4). It was lowest in the South-South (66%). Those with severe multidimensional deprivations were highest in the two Northern regions–North East (40%) and North West (38%) and lowest in the South-South (17%) and South West (23%). In addition to the huge share of the urban-dwelling older persons in multidimensional deprivations, about 20%–22% are still vulnerable to deprivation across the regions, except the North East with the least (14%) and South-South with the highest (30%).

Across the regions, severe multidimensional deprivations were higher among older women than men. In the North East and North West in particular, about half of the women, 52% and 50% respectively, were severely deprived compared to men, 34% and 32% respectively. In the North Central and South East, the proportion of severely deprived women almost doubled that of men. Though women in the South-South and South West are not as severely deprived as their counterparts in other regions, the gender disparity is highest in the two regions, with over 150% and 220% higher share among women than men. However, more men are vulnerable to multidimensional deprivation than women.

Generally, of the five domains of deprivations, health (26.4%), education (25.1%) and living standard (22.2%) contributed the largest share to the multidimensional deprivations of older persons in Nigeria (Table 5). At the sub-national level, education had the largest share of multidimensional deprivation in the North East (30.3%) and North West (31.0%), while health had the largest share in the South-South (35.7%). Deprivation in information/communication was the least across the regions except in the South-South where education was the least in the arrays of older persons’ deprivations. From a gender perspective, overall, males were deprived most in health (29.4%) while women were deprived most in education (26.5%). This gender pattern is reflected at the sub-national level, except in the South-South where older males (38.2%) and females (32.8%) were more deprived of health than in any other dimension, and in the North West where both males (31.0%) and females (31.1%) were deprived most in education.

**TABLE 5:** Contribution of each domain to multidimensional deprivation index (R_0_) at national and subnational levels and by gender (Nigeria, 2023).

	Health (%)	Education (%)	Living standard (%)	Information/Communication (%)	Empowerment (%)	Total (%)
Overall						
Nigeria	26.4	25.1	22.2	9.5	16.8	100.0
Regions						
North Central	27.3	25.4	21.2	10.9	15.3	100.0
North East	25.9	30.3	18.7	9.8	15.4	100.0
North West	24.9	31.0	17.8	11.2	15.1	100.0
South East	25.4	25.2	20.3	7.8	21.3	100.0
South South	35.7	9.2	27.5	10.5	17.1	100.0
South West	25.3	25.0	25.6	9.3	14.8	100.0
Male						
Nigeria	29.4	23.7	23.0	8.3	15.6	100.0
Regions						
North Central	31.0	23.2	22.1	9.8	14.0	100.0
North East	28.1	28.9	19.5	9.4	14.0	100.0
North West	24.5	31.0	17.1	11.6	15.8	100.0
South East	28.6	22.9	21.6	6.9	20.0	100.0
South South	38.2	6.4	30.0	9.3	16.1	100.0
South West	30.8	23.1	25.8	7.3	13.0	100.0
Female						
Nigeria	23.5	26.5	21.3	10.7	18.0	100.0
Regions						
North Central	23.2	26.6	20.4	13.0	16.8	100.0
North East	26.9	31.0	17.3	9.9	15.0	100.0
North West	24.5	31.1	18.3	11.2	14.9	100.0
South East	23.3	27.0	18.6	8.8	22.4	100.0
South South	32.8	12.9	24.0	12.1	18.1	100.0
South West	19.8	28.2	23.0	12.2	16.7	

The results in Table 6 indicate the multilevel multinomial logistic regression estimates of individual-level and community-level factors associated with the risk of multidimensional deprivation among older persons in urban areas of Nigeria. The fixed-effect results indicate that respondents’ age, sex, and household headship were associated with their risk of multidimensional deprivation. For instance, the relative risk ratio (RRR) of being multidimensionally deprived was higher by 90%–143% for females (RRR = 1.90; *p* < 0.001; 95% C.I. = 1.56–2.31) compared to males, and for respondents of age 70–79 (RRR = 1.35; *p* < 0.01; 95% C.I. = 1.14–1.60) and age 80 or above (RRR = 2.43; *p* < 0.001; 95% C.I. = 1.83–3.24) compare to their counterparts below age 70 years (Model 1). However, the risk was about 30% lower for older persons living in households headed by someone else (RRR = 0.70; *p* < 0.001; 95% C.I. = 0.55–0.89) relative to older adults who were themselves the household heads. The associations were consistent when adjusted for other factors in Model 3. The associations between the individual-level factors and risk of severe MD were similar to those of MD. In addition, the gender of the household head was such that older persons living in female-headed households had a lower risk of severe MD, relative to their counterparts living in male-headed households.

**TABLE 6:** Multilevel multinomial logistic regression of predictors of multidimensional deprivation among older persons in urban areas (Nigeria, 2023).

	(Empty model) (outcome variable only)			Model 1 (Individual variables)		Model 2 (Community variables)		Model 3 (Full model)	
	MD	Severe MD	MD		Severe MD	MD	Severe MD	MD	Severe MD
Fixed effects			RRR (95% C.I.)		RRR (95% C.I.)	RRR (95% C.I.)	RRR (95% C.I.)	RRR (95% C.I.)	RRR (95% C.I.)
Sex									
Male ^RC^			1.00		1.00			1.00	1.00
Female			1.90 (1.56–2.31)***		5.70 (4.53–7.18)***			1.90 (1.57–2.32)***	6.18 (4.91–7.78)***
Age group									
<70 ^RC^			1.00		1.00			1.00	1.00
70–79			1.35 (1.14–1.60)**		2.13 (1.73–2.61)***			1.35 (1.14–1.61)**	2.15 (1.75–2.64)***
80+			2.43 (1.83–3.24)***		6.59 (4.81–9.01)***			2.45 (1.84–3.26)***	6.85 (5.01–9.36)***
Marital Status									
Non-widows ^RC,b^			1.00		1.00			1.00	1.00
Widows			0.97 (0.57–1.67)		0.63 (0.34–1.16)			0.96 (0.56–1.65)	0.57 (0.31–1.06)
Household size			0.94 (0.79–1.11)		0.85 (0.69–1.04)			0.94 (0.80–1.11)	0.84 (0.68–1.03)
Household head									
Self ^RC^			1.00		1.00			1.00	1.00
someone else			0.70 (0.55–0.89)**		0.40 (0.30–0.52)***			0.70 (0.55–0.89)**	0.37 (0.28–0.50)***
Sex of household head									
Male ^RC^			1.00		1.00			1.00	1.00
Female			0.84 (0.71–1.00)		0.67 (0.54–0.83)***			0.88 (0.73–1.05)	0.80 (0.65–1.00)^a^
Community-level education									
Low ^RC^						1.00	1.00	1.00	1.00
Middle						0.61 (0.41–0.90)^a^	0.40 (0.25–0.63)***	0.59 (0.40–0.88)^a^	0.37 (0.23–0.60)***
High						0.50 (0.33–0.76)**	0.21 (0.12–0.35)***	0.51 (0.33–0.80)**	0.24 (0.14–0.41)***
Community-poverty									
High ^RC^						1.00	1.00	1.00	1.00
Middle						0.98 (0.65–1.48)	1.35 (0.81–2.24)	1.00 (0.63–1.58)	1.23 (0.71–2.13)
Low						1.02 (0.66–1.57)	1.01 (0.59–1.74)	1.11 (0.67–1.84)	1.15 (0.62–2.12)
Region of residence									
South-South ^RC^						1.00	1.00	1.00	1.00
South West						1.33 (1.01–1.76)^a^	1.90 (1.27–2.83)**	1.32 (0.99–1.76)	1.85 (1.23–2.79)**
South East						1.31 (0.97–1.77)	1.69 (1.10–2.58)^a^	1.35 (0.99–1.84)	1.86 (1.21–2.87)**
North Central						1.33 (0.95–1.86)	1.56 (0.97–2.50)	1.34 (0.95–1.90)	1.62 (1.00–2.63)^a^
North East						1.92 (1.23–2.99)**	3.18 (181–5.58)***	2.23 (1.41–3.52)**	5.34 (3.00–9.51)***
North West						1.18 (0.83–1.69)	2.27 (1.41–3.67)**	1.30 (0.90–1.87)	3.32 (2.03–5.44)***
Random effects									
Variance (SE)	0.35 (0.076)	1.26 (0.168)	0.37 (0.078)		1.01 (0.159)	0.28 (0.069)	0.75 (0.127)	0.31 (0.073)	0.71 (0.131)
VPC (%)	9.6	27.7	10.1		23.5	7.8	18.6	8.6	17.8
Explained variation—PCV (%)	Ref	Ref	5.7		15.2	18.4	33.0	10.4	35.9

^a^ *p* < 0.05; **p < 0.01; ***p < 0.001; SE: standard error, VPC: variance partition coefficient, PCV: proportional change in variance, RC: reference category.

The result of the association between MD and community-level variables indicates that higher community-level education is significantly associated with a lower risk of MD and Severe MD in Models 2. As the community education level goes from low to high, the risk decreases. Also, the region of residence is a significant community-level factor contributing to the risk of MD among older persons in Nigeria. In Model 2, different regions show different risk levels. For instance, individuals from the South West and North East regions have a significantly higher risk of MD compared to the reference group (South-South). However, when adjusted for other factors in Model 3, only the North East showed a consistent association. For severe MD, all the regions except North Central had a higher risk compared to the reference group. The result was consistent and also included the North Central when adjusted for other factors in Model 3.

The result of the random effect indicated the variances attributable to the different nested levels in the model. The empty model which contains no explanatory variable indicated that between-communities variation, expressed as VPC, associated with the risk of severe MD (27.7%) was larger than between-community variation associated with the risk of MD (9.6%), and this gap was consistent across individual and community levels. The result further indicated that intra-community variation associated with the risk of MD and severe MD was estimated at 18.4% and 33.0% respectively (Model 2), and when adjusted for other factors was estimated at 10.4% and 35.9% respectively (Model 3). These results indicated that community-level factors account for a large proportion of the variation in the risk of multidimensional deprivations and severe multidimensional deprivations.

## Discussion

Older people in low- and middle-income (LMIC) settings like Nigeria are increasingly at higher risk of poverty compared to other population groups. This is partly due to the prevailing economic crisis in the country. This study examines the level of MD and associated factors among older people in urban geographies of Nigeria. The findings from this study serve as a reference for developing community-based interventions to improve the welfare and wellbeing of older people in urban areas of the country.

The results presented in this study provide valuable insights into the MD experienced by older adults in the urban setting of LMIC, taking Nigeria as a case study. The findings reveal an alarming level of headcount MD among this population, with over 75% being multidimensionally deprived among which 27% were severely deprived. The rate among older persons implies that approximately seven million urban-dwelling older persons in Nigeria are experiencing severe deprivation across multiple dimensions. An additional 23% of older persons were vulnerable to multidimensional deprivation. This poses a big challenge to the wellbeing and quality of life of older people, indicating the extent of the problem and the need for urgent attention and targeted interventions to address various domains of deprivation. This level of MD is higher than the rate reported by the National Bureau of Statistics (NBS) for under-five children (70%), the highest rate among population groups in Nigeria [28]. It is also far higher than the prevalence in high-income countries like the United States [12] with a 12% prevalence, and some developing countries like Iran with a 59% prevalence [29]. Of all the dimensions considered, health (26%), education (25%) and standard of living (22%) contributed the largest share to the MD. The contributions of these indicators align with the 2015 AgeWatch index in which Nigeria was ranked one of the lowest globally in health index, level of education and socioeconomic situations of older persons [30].

The headcount and intensity of MD provide a more comprehensive insight into the condition of older persons in Nigeria, unlike other studies which have utilised only income or socioeconomic status to measure poverty among older people [4]. The intensity of deprivation among the severely deprived individuals, with an average deprivation score of over 69% of the indicators and among the multidimensionally deprived group, with an average deprivation score of 50%, is particularly of serious concern. It underscores the comprehensive nature of the deprivation experienced and reinforces the need for multifaceted interventions that address various dimensions of the deprivation simultaneously. Though a few existing studies in Nigeria have delved into poverty among older people [4, 31], none has indicated the extent of poverty among the poor, as revealed in this study.

The study also reveals significant gender inequalities in multidimensional poverty levels among older adults in Nigeria. Women, in particular, face higher levels of severe deprivation compared to men, with the MDI for women almost double that of men. This disparity reflects women’s limited access to education, lower levels of participation in household economic and health decision-making, and limited access to employment opportunities [22]. The gender disparity is evident across various regions, with Northern women experiencing the worst condition of severe MD. This highlights the need for gender-sensitive interventions to address the specific challenges faced by older women in accessing resources and opportunities.

Furthermore, this study finds that living in a household headed by someone else, especially by a family member, protects older persons from MD. Although being a household head could give older persons some authority over household members and resource allocation [32], this benefit is conditional on the availability of resources, the extent of affinity, sense of responsibility and reciprocity, and the functional ability of the household head. Thus, older persons living in households headed by a younger, economically active and financially buoyant family member are more likely to have access to information, communication and companionship, healthcare support and quality living standards, compared with those with otherwise living arrangements. However, if the household is headed by the spouse, it may not benefit some older women who are economically vulnerable and marginalised in household headship and resource sharing in a patriarchal society [33, 34].

The subnational decomposition analysis provides further valuable insights into the regional disparities in multidimensional deprivation. The North East and North West regions exhibit higher rates of deprivation, with a significant proportion experiencing severe deprivation. The two regions have often shown the worst case of many socioeconomic and health indicators including the level of education, healthcare service utilisations, unemployment, multidimensional deprivations in general and sub-groups of the population [22, 28, 35]. The situation in Northern regions may also be linked to the women’s low level of education, restriction in some public spaces and economic activities, and culturally laden norms that limit women’s economic and social power in the regions [36]. On the other hand, the South-South region, though with a lower level of headcount MD, reports the highest vulnerability to multidimensional deprivation. These regional variations emphasize the importance of tailoring interventions to address the unique challenges and context of each region, considering factors such as socioeconomic conditions, healthcare infrastructure, and cultural factors.

The multilevel regression analysis identifies individual-level and community-level factors associated with the risk of multidimensional deprivation among urban-dwelling older persons in Nigeria. This study found that the age of the respondents was a major individual-level factor influencing the risk of multidimensional deprivation. In particular, older persons, especially women, face a higher risk of multidimensional deprivations. This finding may be because individuals at advanced ages are more vulnerable to poverty due to their reduced participation in economic activities coupled with caregivers’ financial incapacity [37, 38] and poor socioeconomic conditions in the country [39]. However, the finding contrasts the that of Dhongde [40] who linked lower multidimensional deprivation to older age. This disparity may be because Dhongde’s evidence was based on a high-income context.

The study, however, found that those living in communities with high levels of education experience a lower risk of deprivation. These findings underscore the importance of not only considering individual characteristics but also recognizing the broader context of the community when addressing health outcomes. This has implications for designing interventions and policies that aim to reduce the prevalence of severe MD. Meaning impact could be achieved when community-level factors are given the necessary focus.

### Potential Health Implications

The findings of this study have significant health implications. The huge proportion of multidimensional deprivations suggests that a significant portion of older adults lack access to essential elements of wellbeing, including health services, education, standard living conditions, and social support. Specifically, individuals with deprivation in the health domain, for instance, are liable to functional limitations which can be worsened in the absence of relevant support [40]. This potentially increases older people’s risk of hospitalisation and health expenditure. A similar implication is inferable for education, as more than half of older persons are uneducated and will be susceptible to adverse health outcomes associated with a low level of education. Also, living arrangements, access to information and communication devices are key indicators of information and communication in this study. Older persons living alone are at risk of loneliness and depression [41]. In the same vein, lack of access to information, either from radio, television or mobile phones, results in a lack of access to health information which has implications for older persons who need regular healthcare services. Poor standard of living puts older people at risk of exposure to infectious diseases amidst their declining immunity.

The gender disparities also stress the need for gender- and situation-specific interventions to improve the health and overall wellbeing of older adults, especially women, in urban areas. Furthermore, from a community-level point of view, structural inequalities need to be addressed to enhance the health outcomes of older populations, promote equitable access to healthcare, and improve their overall quality of life.

### Limitations to the Study

The study utilized a cross-sectional design, which limits its ability to establish causal relationships. Longitudinal data would be necessary to better understand the dynamics of multidimensional deprivation over time. Besides, variables and measures in this study were adapted to the extent of data availability; thus, it might not fully capture all dimensions of multidimensional deprivation that could be relevant to the older population. However, the dimensions and indicators used are comprehensive and arguably cover the major socioeconomic and health concerns of older persons. The construction of the multidimensional deprivation index involved assigning weights to indicators. Though different weighting methods could lead to different conclusions, I do not envisage any major difference in the conclusions. Also, the choice of a cut-off value (40%) to define multidimensional deprivation was based on considerations for poverty levels tolerable in an LMIC context, but different cut-off points could yield different estimates of deprivation. However, divergent estimates do not in any way invalidate the estimate of this study, as long as it is interpreted within its premised assumptions. This study focused on urban areas, which might limit the generalizability of the findings to rural settings or other countries with different contexts and socioeconomic conditions.

### Conclusion

This study indicates an alarming prevalence of multidimensional deprivations among urban-dwelling older adults in Nigeria, though gender disparities are evident, with women more severely deprived. It highlights that the deprivations were driven, not only by individual factors but also by community-level factors, including education and region of residence. The findings of this study underscore the urgent need for comprehensive and targeted policy interventions to address the multidimensional deprivation faced by older adults in urban Nigeria. These interventions should prioritize addressing gender disparities, targeting regions with high deprivation rates, and considering individual and community characteristics. By addressing the underlying determinants of multidimensional deprivation and promoting health equity, policymakers and stakeholders can work towards improving the wellbeing and quality of life for older adults in urban Nigeria.
